# Coxa Vara

**Published:** 1908-06-20

**Authors:** 


### COXA VARA.

Coxa Vara is a name which was originally applied to a condition in which a well-defined deformity of the femur makes its appearance for no apparent reason. In its typical form the disease so described is unilateral and the changes in the bone are confined to its upper end. They are (1) an alteration in the relation of the axis of the neck to that of the shaft so that the angle between the two may be reduced to a right angle or even less, and (2) an antero-posterior curve in the neck itself, which becomes convex on its anterior aspect.

The clinical results of such a deformity are at once apparent. The limb is externally rotated and adducted towards the middle line. There is, of course shortening of the limb and the upper limit of the great trochanter will be found to be above Nélaton's line. The patients suffer from a double inconvenience; the adduction of the limb impedes progression because the affected limb comes into contact with and knocks against the sound one at each step, and abduction is impossible beyond a certain degree, the limitation being caused by the upper surface of the great trochanter coming against the crest of the ilium.

The amount of disability which results varies, of course, with the degree of deformity present. It may be so slight as to be barely noticeable, or on the other hand so serious as to render progression altogether impossible without crutches. In some cases the deformity is bilateral, giving rise to the so-called "scissor-leg" deformity. If this be the case the patient either cannot walk at all or does so with a waddling gait, which is so characteristic that the lesion can be diagnosed at sight.

In its true form it is said to be due to adolescent rickets, although the association of rickets in childhood with coxa vara subsequently has not been definitely established; and there are certain difficulties in the way of accepting the statement absolutely. One might ask, for instance, why rickets coming on in adolescence should attack the neck of the femur, which is not a place where much growth of bone takes place.

In other deformities due to rickets the association is easier to account for. In genu valgum, for instance, the softened bone is compressed on the outer side, through which the weight of the body is transmitted, and grows unduly on the inner side, which is free from such restrictions. The internal lateral ligament stretches secondarily and becomes inadequate, and so the genu valgum is produced. A similar argument might, it is true, apply to coxa vara, that the bone becomes softened by rickets and that the neck of the femur, so to speak, collapses under the weight of the body and its angle is reduced. But this does not explain the antero-posterior curve in the neck which is also present, unless we are to suppose that the softening allows the external rotator group to exert their power to the fullest advantage and thus bend the bone. The truth of the matter is that coxa vara is a mysterious affection whose ætiology is far from clear.

Recently, however, it has become the custom to classify under the heading of coxa vara a number of conditions in which the cause is known, but in which the deformity is not the typical one. The most important group of cases which has thus been introduced is that in which an alteration has been produced in the upper end of the femur by some form of injury. The systematic application of $x$-rays to cases which resemble true coxa vara has revealed the fact that in a great number an irregularity in the line of the neck could be demonstrated. The explanation put forward is that either the upper epiphysis has been separated or the neck of the bone fractured and union in malposition has supervened.

Such an injury may easily be overlooked in a child, especially if it takes place before the child has begun to walk, because the violence which is necessary to produce it is astonishingly slight, and the injury is either painless, or if it causes pain the child is unable to call attention to it. Such a case came under the author's observation quite recently. A child aged two was noticed by its mother to keep its leg in an abnormal position. She found this when she was bathing it. It had not been fretful nor complained of pain. The mother could remember no accident having happened to it. The right leg was shortened and strongly adducted, so that the axis of the right femur lay across the sound one at the junction of its middle and lower thirds. The great trochanter was above Nélaton's line. But the leg was internally rotated as well as adducted, and it was obviously therefore not a typical case of coxa vara. It gave the appearance more of being a dorsal dislocation of the hip. The $x$-rays showed that the deformity was due to a separation of the head of the femur from the neck, which had then slipped up and united in the new position; and yet, though one knew this, further inquiry failed to elicit from the mother any remembrance that the child had been dropped or injured.

Similar cases could be quoted. Can one classify them as coxa vara? All one can say is that there is an alteration in the upper end of the femur due to injury. The deformity produced is not typical of coxa vara, because there is no curvature of the neck of the bone, although there may sometimes be external rotation; because when the neck of the femur is fractured the limb is everted by its own weight, and union may take place in this position.

Other conditions which have been said to cause coxa vara are achondroplasia, cretinism, and osteoarthritis. In the latter disease the head of the femur becomes definitely changed in shape so that the neck of the bone assumes a higher position relatively to the acetabulum, and the great trochanter may become so enlarged by osteophytic outgrowths that its upper surface lies above Nélaton's line, and abduction is limited by the osteophytes coming in contact with the surface of the ilium. The effects so produced may resemble a true coxa vara, but the pathological process is an essentially different one.

If all these conditions are to be classified under the heading of coxa vara that title must be regarded as defining a collection of physical signs more or less similar but due to different causes and not as representing a discriminate affection.

